# Efficacy of the LED Red Light Therapy in the Treatment of Temporomandibular Disorders: Double Blind Randomized Controlled Trial

**DOI:** 10.1155/2019/8578703

**Published:** 2019-05-06

**Authors:** Ahmed Fadhel Al-Quisi, Auday M. Al-Anee, Hassanien A. AL-jumaily, Eman F. Bahr, Dina A. Finjan

**Affiliations:** ^1^Oral and Maxillofacial Surgery Department, College of Dentistry, University of Baghdad, Iraq; ^2^Al-Kindy Teaching Hospital, Baghdad, Iraq; ^3^Al-Shaheed Gazi Al-Hariri Teaching Hospital, Medical City, Baghdad, Iraq; ^4^Resident Dentists at the Ministry of Health in Baghdad, Iraq

## Abstract

**Background:**

Temporomandibular dysfunction syndrome (TMD) is a common disease among dental patients. It occurs as a consequence of malfunction of the tempromandibular and/or surrounding facial muscles. LED red light therapy is not been well established, and it is important to find out the role of this technique in the treatment of temporomandibular disorders.

**Aim of the Study:**

To evaluate the efficacy of the LED red light in the treatment of the tempromandibular dysfunction syndrome.

**Material and Methods:**

Fifty students of the College of Dentistry/University of Baghdad with myofacial pain associated with Tempromandibular Disorder volunteered to participate in this study and be evaluated during both treatment and follow-up periods. They were 40 (80%) females and 10 (20%) males. Patients were divided into 2 groups: Group A treated by TenDlite® Medical Device model 204 with a LED's of wavelength 660 nm (red light) and Group B given placebo (no treatment at all) by just putting the TenDlite device near the tender points without battery and turning ON the device.

**Results:**

The changes in the pain value and number of the tender muscles in both groups were highly significant, only placebo group less but with no significant differences.

**Conclusions:**

This study showed that red LED therapy could be useful in improving patient's symptoms regarding pain, clicking, and number of tender muscles. In addition, this study showed the importance of the psychological part of treatment of those patients. This trial is registered with TCTR20190406002.

## 1. Introduction

Temporomandibular (TMJ) dysfunction syndrome usually refers to a group of symptoms, which may include pain, clicking, and limitations of the mandibular movement [1, 2].

However, the exact pathophysiology of the Temporomandibular Dysfunction Syndrome is not entirely understood. It is believed to be a multifactorial disease [2, 3].

Local insults and systemic disorders could be involved in the development of Temporomandibular Dysfunction Syndrome. Local insults include articular disc displacement and abnormal joint structure that resulted from different conditions like hypoplastic mandibular condyles, rheumatoid arthritis, clenching, and bruxism, and local trauma also play a significant role [3].

Stress, age, and sex are considered of the most important risk factors for the development of Temporomandibular Dysfunction Syndrome. As stress induces muscle hyperactivity which is followed by muscle fatigue, the end result will be contracture of the muscle, occlusal disharmony, internal derangement, and degenerative arthritis [4].

Different modalities have been applied for treatment of Temporomandibular Dysfunction Syndrome, but without specifying the best or optimal method of treatment [5].

Usually dentistry undergraduate students suffer from tremendous psychological stresses of exams, practical and clinical requirements, and dealing with patients for the first time.

This study included only students of preliminary studies at the College of Dentistry, University of Baghdad, who have myofacial pain and clicking associated with Temporomandibular Dysfunction Syndrome.

The aim of this study is to evaluate the efficacy of the LED (light Emitting diode) red light in the treatment of the tempromandibular dysfunction syndrome.

## 2. Patients and Methods

This is an Interventional, Case Control, Randomized Double Blind, Comparative Clinical Study that was conducted in the Department of Oral and Maxillofacial Surgery at the College of Dentistry/University of Baghdad, between December 2017 and May 2018.

This study was carried out according to the ethical principles and in compliance with the Declaration of Helsinki.

All of the participants volunteered to be part of this research were students of the Faculty of Dentistry at University of Baghdad and all of them have signed the free and informed consent term.

Fifty students with TMD symptoms were enrolled in this study, 40 (80%) patients were females and 10 (20%) patients were males. The diagnosis of TMD was based on history and clinical examination.

All patients with history of trauma to the joint, orthodontic treatment, or previous treatment for the same symptoms in the last 6 months were excluded from this study.

A history was taken from each patient regarding the following: age, sex, habits, history of stress factors, dental treatment received and duration of symptoms, TMD treatment history, and current medication/appliance.

Physical examination was performed for each patient regarding the following: pain and tenderness in the jaw muscles (by palpation), clicking or noises in the joint (by auscultation), amount of the mouth opening (distance between the incisal edges of upper and lower incisor teeth).

**Figure 1:**
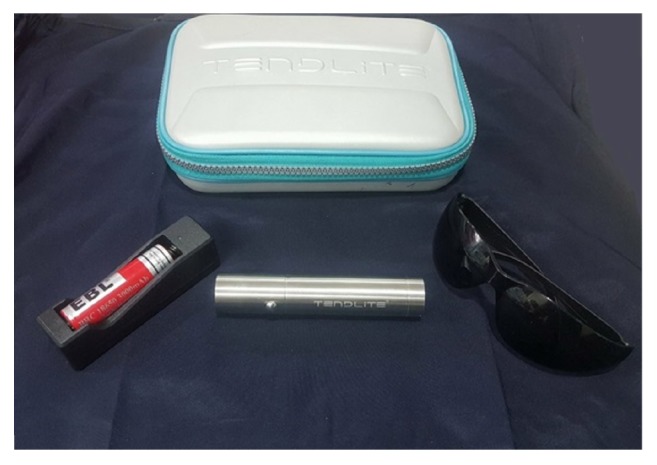
TenDlite LED red light device with accessories.

All patients had received no treatment for their condition at least 2 months before enrolling in the study. A consent was taken from each patient before starting the trial and after a full explanation of the nature of the syndrome, possible causes, prognosis, different treatment options, and the possible side effects for each type of treatment. TenDlite® Medical Device model 204 with a LED's of wavelength 660 nm (red light) has been used according to manufacturer instructions. The optical power of the TenDlite is 1.6 watt, which is equivalent to 1.6 joules of work per second (Figure 1).

Patients were divided into 2 groups, according to the type of therapy.

*Group A*. Twenty-five patients were included in this group. 18 (72%) patients were females and 7 (28%) were males; these were given LED red light to the affected TMJ with lateral pterygoid muscle of the same side; further light may be given to the tender points of masseter and temporalis muscles if present.

The LED red light was applied for 3 minutes for each point according to the manufacturer instructions.

*Group B*. Twenty-five patients were included in this group. 22 (88%) patients were females and 3 (12%) were males; these were given placebo (no treatment at all) by just putting the TenDlite device near the TMJ and other tender points without placing any battery in the device.

Permuted block of randomization with Microsoft Excel (2013) was used in this study to avoid the imbalance of the number of participants in two groups.

Treatment of both groups continued for four weeks (one session given each week with total of 4 sessions).

All patients were evaluated by the same Independent Maxillofacial Surgeon clinically by looking for any changes in the pain and tenderness in the jaw muscles, clicking or noises in the jaws, amount of the mouth opening and headache in each visit.

Visual analogue scale has been used for pain assessment (pain measured by a scale from zero to 10, where zero represents the lowest pain value and 10 the highest).

The participants enrolled in this study were surgeon who evaluated the response to the treatment and the statistician not knowing the type of treatment delivered to the patients.

Independent T Test with Dunn-Sidak adjustment was used for statistical analysis.

Differences are statistically significant if P value ≥ 0.05.

## 3. Results

Fifty students of the College of Dentistry/University of Baghdad with TMD symptoms volunteered to participate in this study and they were evaluated during both treatment and follow-up periods. They were 40 (80%) females and 10 (20%) males, whose age ranged from 19 to 24 years with mean age 20.7 years.

In this study 85% of the patients thought that stress associated with daily exams in the college is the main cause of their symptoms, 7% attributed it to the trauma from eating hard food, 5% linked it to orthodontic treatment, and 3% was thought to be caused by depression.

Symptoms were found to be worse with yawing in 45% of the patients, whereas 30% of those patients found it worse with chewing gum and 25% of them suffered severe symptoms after eating hard food.

TMD affected left side of the face in 90% of the cases and the symptoms were progressively worsening in all patients.

There was no statistical significant difference in demographic features between the two groups (Table 1).

All the patients enrolled in this study had normal range of mouth opening and no changes were observed in the treatment sessions for both groups.

TMJ clicking resolved in all of Group A patients, and only three patients got improvement in Group B.

On comparing the effect size of both groups, both groups had a medium size of effect when comparing the pain score in the 2nd visit with the baseline visit with large size of effect in both groups from the 3rd visit, with a larger value noticed for Group A (Table 2).

Regarding the number of tender muscle reduction. The effect size is large form the 2nd visit in Group A patients but in Group B it reaches large value only at the 4th visit (Table 3).

## 4. Discussion

Although this study applied LED red light therapy for TMD treatment, LEDs with higher output power (about 3 Watts) have been used without side effects for different dental procedures, such as photo activation of dental bleaching gel [6] and curing of composite resins [7].

In the current study, we used similar average safe parameters of LED red light therapy that have been used in the previous studies, which were conducted on ex vivo specimens [8].

Generally, previous studies have reported a higher prevalence of TMD in females than in males [6, 7]. Although these differences have been explained by behavioral, psychosocial, hormonal factors, no clear conclusions have been drawn [9, 10]. In our study, females were found to be at a higher risk of TMD than males with highly significant differences between them (p value of 0.0001).

The action of the red light begins when it is absorbed by tissues. The absorbed energy then will be dissipated into heat [8].

These thermal effects of the LED red light therapy may clarify the results of treatment, because phototherapy can lead to vasodilation with increased blood and oxygen supply and transport and this led to washout of all inflammatory mediators in the target area as has been proven in several previous studies [11–15].

In addition, several studies [16, 17] have shown that nerve conduction velocities can be altered by phototherapy, which result in analgesic effects [17].

All these facts are mentioned previously in the literature and it may give a good explanation to the statistically highly significant improvement in patients' symptoms regarding pain and also the number of the tender muscles in Group A patients who managed with LED red light therapy from the baseline visit to the 4th visit.

This part of the research results may give an impression that LED red light therapy method is very effective in treating TMD cases. However, if we complete the discussion of the results of Group B patients, it may become clear that a large part of the results of Group A patients have been affected by the psychological state of the patient, because of the psychological distresses present in all Group A patients and in 90% of the Group B patients; this result also shows us how the psychological factors are highly important in the development of the TMD [18].

In addition, patients' expectancy about the new treatment modality could result in higher hopes for better treatment results, which may shape neuronal activity as if any other specific sensory stimulation were present, resulting in a complex blend of different support physiologies [19], constituting a secondary effect [20]. That might have led to an improvement in patients' symptoms.

All these previous facts may influence our results with Group B patients who have been treated only with placebo (no treatment at all) by just putting the TenDlite device near the tender points without placing any battery in the device.

Surprisingly, three patients in Group B complained from clicking that disappeared after two to three sessions of the placebo treatment.

However, last two tables show that both groups show significant improvement in aspects of pain and number of tender muscles, but the LED red light group shows a better results than placebo group especially in reduction of the number of tender muscles.

## 5. Conclusions

This study showed that red LED therapy could be useful in improving patient's symptoms regarding pain, clicking and number of tender muscles. In addition, this study showed the importance of the psychological part of treatment of those patients.

## Figures and Tables

**Table 1:** Showing demographic features of patients in both groups.

Parameter		Group A	Group B	P value
Gender	Female	18 (72%)	22 (88%)	
	Male	7 (28%)	3 (12%)	

Age	Mean	21.8	19.5	0.0001

Psychological distress		100%	90 %	

Duration of the symptoms in years (average)		1.4	1	0.6868

Previous Treatment		60%	20%	

Degree of pain before starting treatment *∗* (mean)		7.2	5.56	0.0066

Habit history		60%	40%	

Presence of clicking		80%	80%	

*∗*: pain measured by a scale from zero to 10, where zero represents the lowest pain value and 10 the highest.

**Table 2:** Comparison of the effect size between group A and B in pain reduction.

Periods	Effect size	Effect size
	Group A	Group B
Base line visit	0.56874	0.617133
2nd visit		

Base line visit	1.23359	0.993695
3rd visit		

Base line visit	1.956287	1.204716
4th visit		

^∧^=Dunn-Sidak adjustment, effect size=0.2 small, 0.5 medium, 0.8 large.

**Table 3:** Comparison of the effect size between group A and B in number of tender muscles reduction.

Periods	Effect size	Effect size
	Group A	Group B
Base line visit	1.103711	0.56874
2nd visit		

Base line visit	2.363637	0.717107
3rd visit		

Base line visit	3.353919.	1.050685
4th visit		

^∧^=Dunn-Sidak adjustment, effect size=0.2 small, 0.5 medium, 0.8 large.

## Data Availability

The data used to support the findings of this study are included within the supplementary information file “available here”.

## Supplementary Materials

Supplementary Materials This supplementary xle. file contains patients data about pain in the 2 study groups.
